# Hepatitis B virus infection as a neglected tropical disease

**DOI:** 10.1371/journal.pntd.0005842

**Published:** 2017-10-05

**Authors:** Geraldine A. O’Hara, Anna L. McNaughton, Tongai Maponga, Pieter Jooste, Ponsiano Ocama, Roma Chilengi, Jolynne Mokaya, Mitchell I. Liyayi, Tabitha Wachira, David M. Gikungi, Lela Burbridge, Denise O’Donnell, Connie S. Akiror, Derek Sloan, Judith Torimiro, Louis Marie Yindom, Robert Walton, Monique Andersson, Kevin Marsh, Robert Newton, Philippa C. Matthews

**Affiliations:** 1 Faculty of Infectious and Tropical Diseases, London School of Hygiene and Tropical Medicine, London, United Kingdom; 2 Co-infection Studies Programme, MRC/UVRI Uganda Research Unit, Entebbe, Uganda; 3 Nuffield Department of Medicine, Peter Medawar Building for Pathogen Research, Oxford, United Kingdom; 4 Division of Medical Virology, Stellenbosch University, Faculty of Medicine and Health Sciences, Tygerberg, Cape Town, South Africa; 5 Department of Paediatrics, Kimberley Hospital, Kimberley, South Africa; 6 Centre for Infectious Disease Research in Zambia, Lusaka, Zambia; 7 Health System Research Ethics Department, KEMRI Wellcome Trust Research Programme, Kilifi, Kenya; 8 Mother and Child Health Department, Baringo County Referral Hospital, Baringo, Kenya; 9 Medical-Surgical Department, Machakos Level 5 Hospital, Machakos, Kenya; 10 Garissa County Referral Hospital, Garissa, Kenya; 11 Patient and Public Involvement Committee, Translational Gastroenterology Unit, Nuffield Department of Medicine, John Radcliffe Hospital, Oxford, United Kingdom; 12 Global Healthcare Public Foundation, Kampala, Uganda; 13 School of Medicine, Medical & Biological Sciences, University of St Andrews, St Andrews, Scotland, United Kingdom; 14 Chantal Biya International Reference Centre for Research on HIV/AIDS, Yaounde, Cameroon; 15 Faculty of Medicine and Biomedical Sciences, University of Yaounde I, Yaounde, Cameroon; 16 Nuffield Department of Medicine, University of Oxford, Oxford, United Kingdom; 17 Warwick Medical School, University of Warwick, Coventry, United Kingdom; 18 Department of Infectious Diseases and Microbiology, Oxford University Hospitals NHS Foundation Trusts, John Radcliffe Hospital, Oxford, United Kingdom; 19 Africa-Oxford (AfOx) Initiative, Peter Medawar Building for Pathogen Research, Oxford, United Kingdom; 20 Department of Health Sciences, University of York, York, United Kingdom; University of Texas Medical Branch, UNITED STATES

## Background

**Fig 1:**
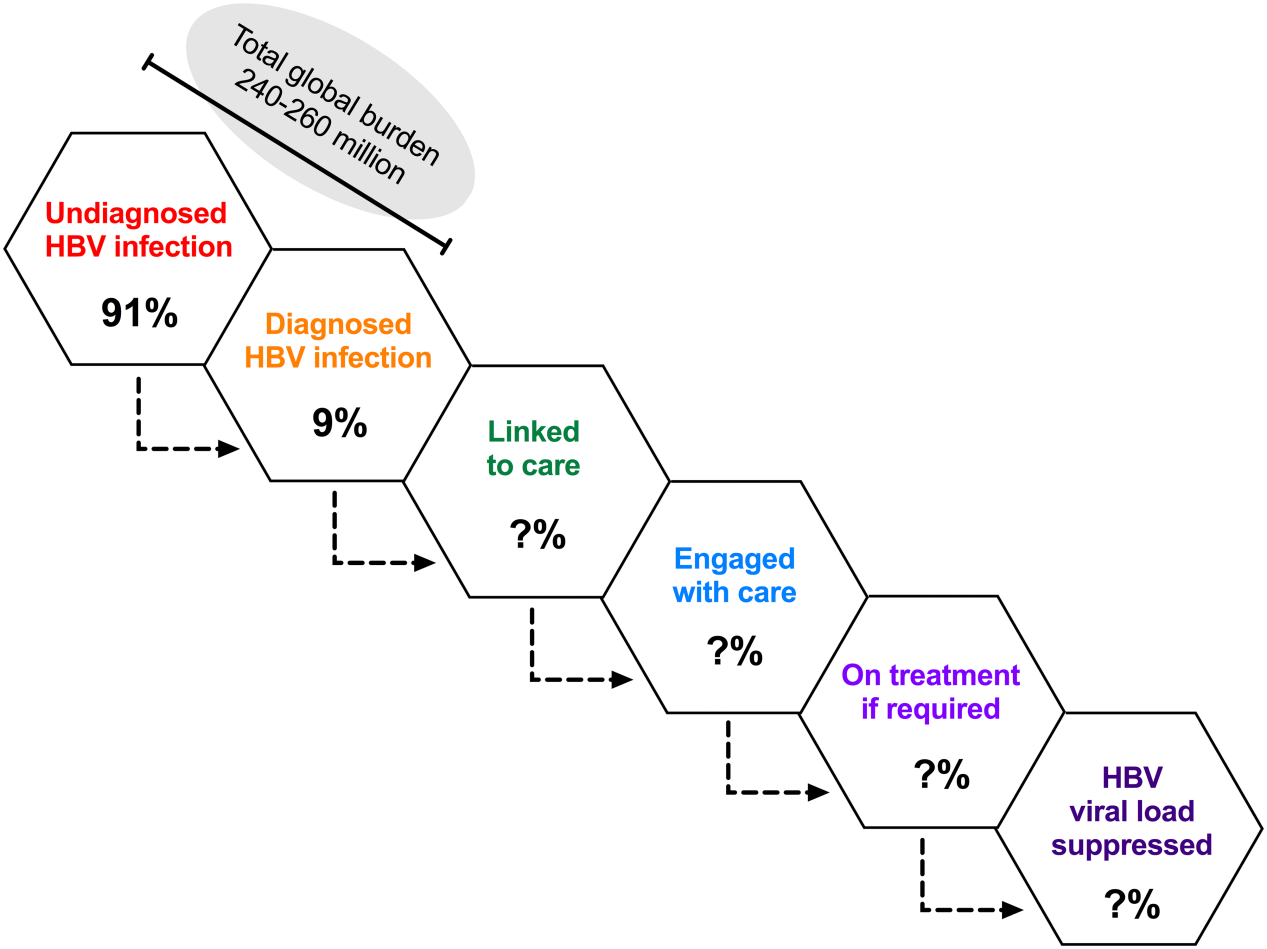
The hepatitis B virus (HBV) cascade. Diagrammatic representation of the total burden of HBV infection and the subsets of individuals who are diagnosed (orange), linked to care (green), engaged with care (blue), on treatment (light purple), and have suppressed viremia (dark purple). An estimate of the proportion of cases undiagnosed versus diagnosed (91% versus 9%, respectively) is based on the WHO fact sheet [3]. The proportion who flow from each pool to the next is otherwise represented by a question mark, as these numbers are not represented by robust data.

The Global Hepatitis Health Sector Strategy is aiming for “elimination of viral hepatitis as a public health threat” by 2030 [1], while enhanced elimination efforts for hepatitis are also promoted under the broader remit of global Sustainable Development Goals (SDGs) [2]. This is an enormous challenge for hepatitis B virus (HBV) given the estimated global burden of 260 million chronic carriers, of whom the majority are unaware of their infection [3] (Fig 1).

We here present HBV within the framework for neglected tropical diseases (NTDs) [4] in order to highlight the ways in which HBV meets NTD criteria and to discuss the ways in which the NTD management paradigm could be used to strengthen a unified global approach to HBV elimination [5]. The major burden of morbidity and mortality from HBV is now borne by tropical and subtropical countries [6]. Many African populations epitomize specific vulnerability to HBV [7], so we here focus particular attention on Africa, both through focus on the existing published literature and through presentation of a unique data set of opinion and experience (see S1 Supporting Information). However, the themes we represent are transferable to other low- and middle-income settings and are relevant on the global stage.

## Current strategies for HBV control

Robust preventive vaccines have been rolled out in Africa since 1995 as a component of the Expanded Programme on Immunization (EPI). Traditionally, most vaccine campaigns have relied upon monovalent HBV vaccines (for summary, see https://doi.org/10.6084/m9.figshare.5242303.v1). For adults with chronic infection and evidence of ongoing liver damage, a daily dose of suppressive antiviral therapy using nucleot(s)ide analogues (Table 1) is successful at mediating viremic suppression in the majority of cases, reducing complications and diminishing spread. Antiviral therapy does not commonly result in cure, due to the persistence of DNA in the hepatocyte nucleus, in the form of both cccDNA and integrated HBV DNA, but interferon (IFN)-based therapy can increase rates of clearance.

**Table 1:** Drug therapy used to treat HBV. Costing is based on the International Medical Products Price Guide: http://mshpriceguide.org/en (data accessed May 2017. Price for lamivudine (3TC)—South Africa Department of Health; Price for tenofovir (TDF)—Supply Chain Management Project; price for HBV immunoglobulin (HBIG)—Sudan Medicins Sans Frontieres). WHO essential medicines: http://who.int/medicines/publications/essentialmedicines/EML_2015_FINAL_amended_NOV2015.pdf?ua=1.

Drug name	Drug class	Potency against HBV*	Resistance	Severe adverse effects	Safe in pregnancy?	Use in children	Use as part of combined ART?	WHO “essential medicine”	Monitoring	Cost (International Medical Products Price Guide)
**Tenofovir (TDF)**	Nucleotide reverse transcriptase inhibitor	+	Rare	Lactic acidosis, hepatitis, renal injury, bone demineralization	Yes	>12 years for HBV**	Yes	Yes	LFTs, renal function	US$3.91/month
**Entecavir (ETV)**	Nucleoside reverse transcriptase inhibitor	++	<10% at 3 years. Increased in 3TC resistance	Lactic acidosis, steatosis	Not known	From age 2 years	No	Yes	LFTs, FBC	Not listed
**Lamivudine (3TC)**	Nucleoside reverse transcriptase inhibitor	+ (potentially limited by resistance)	50% at 3 years. Best-recognized mutations are in YMDD motif in viral polymerase.	Lactic acidosis, hepatomegaly and steatosis, pancreatitis	Yes	From birth	Yes	Yes	LFTs, FBC	US$1.43/month
**Interferon (IFN)**	Biologic response modifier	+ (genotype dependent)	No	Anorexia, diarrhea, flu-like symptoms, neurotoxicity, seizures, hepatotoxicity	No	Not recommended in children (>18 years only)***	N/A	Yes	LFTs, FBC, TFTs	Not listed
**HBV immuno-globulin (HBIG) for prophylaxis**	Biologic response modifier	++	N/A	Abdominal pain, buccal ulceration, chest pain	Yes	From birth	N/A	No	N/A	US$38.02/dose

**Abbreviations:** ART, antiretroviral therapy (for HIV infection); FBC, full blood count; HBV, hepatitis B virus; LFT, liver function test; N/A, nonapplicable; TFT, thyroid function test; YMDD, tyrosine-methionine-aspartic acid-aspartic acid motif.

*Potency against HBV is defined as + or ++ to differentiate between agents with lower and higher suppressive capacity, respectively.

** British National Formulary (https://www.bnf.org/) states tenofovir can be prescribed for HIV in infants >2 years, but data for HBV treatment are lacking.

*** British National Formulary (https://www.bnf.org/) states Peg-interferon-alpha can be prescribed for chronic hepatitis C virus (HCV) in infants >5 years, but data for HBV treatment are lacking. https://www.medicinescomplete.com/mc/bnfc/current/

Prevention of mother to child transmission (PMTCT) can be improved through a combination of routine antenatal screening, antiviral drugs during the latter stages of pregnancy, and HBV vaccination to the baby starting at birth. Where resources permit, HBV immunoglobulin (HBIG) can further reduce the risk of vertical transmission.

Despite the efficacy of these strategies in managing or preventing individual cases, these interventions do not currently offer a route to global HBV eradication, due to a shortage of investment and resources, the large pool of undiagnosed cases, lack of routine diagnostic screening, the high cost of IFN and HBIG, the lack of a curative therapy, substantial gaps in drug and vaccine coverage, and the potential for increasing drug resistance [8].

## Application of NTD criteria to HBV

We have applied the WHO criteria for NTDs to HBV [4] and refer to case studies and experience from our own clinical practice (S1 Supporting Information) to illustrate how HBV in Africa fulfills NTD criteria. Some of the factors underpinning the neglect of this infection are summarized in Table 2.

**Table 2:** Summary of factors potentially contributing to the neglect of investment in hepatitis B virus (HBV) clinical care, research, advocacy, and education.

**Fig 2:**
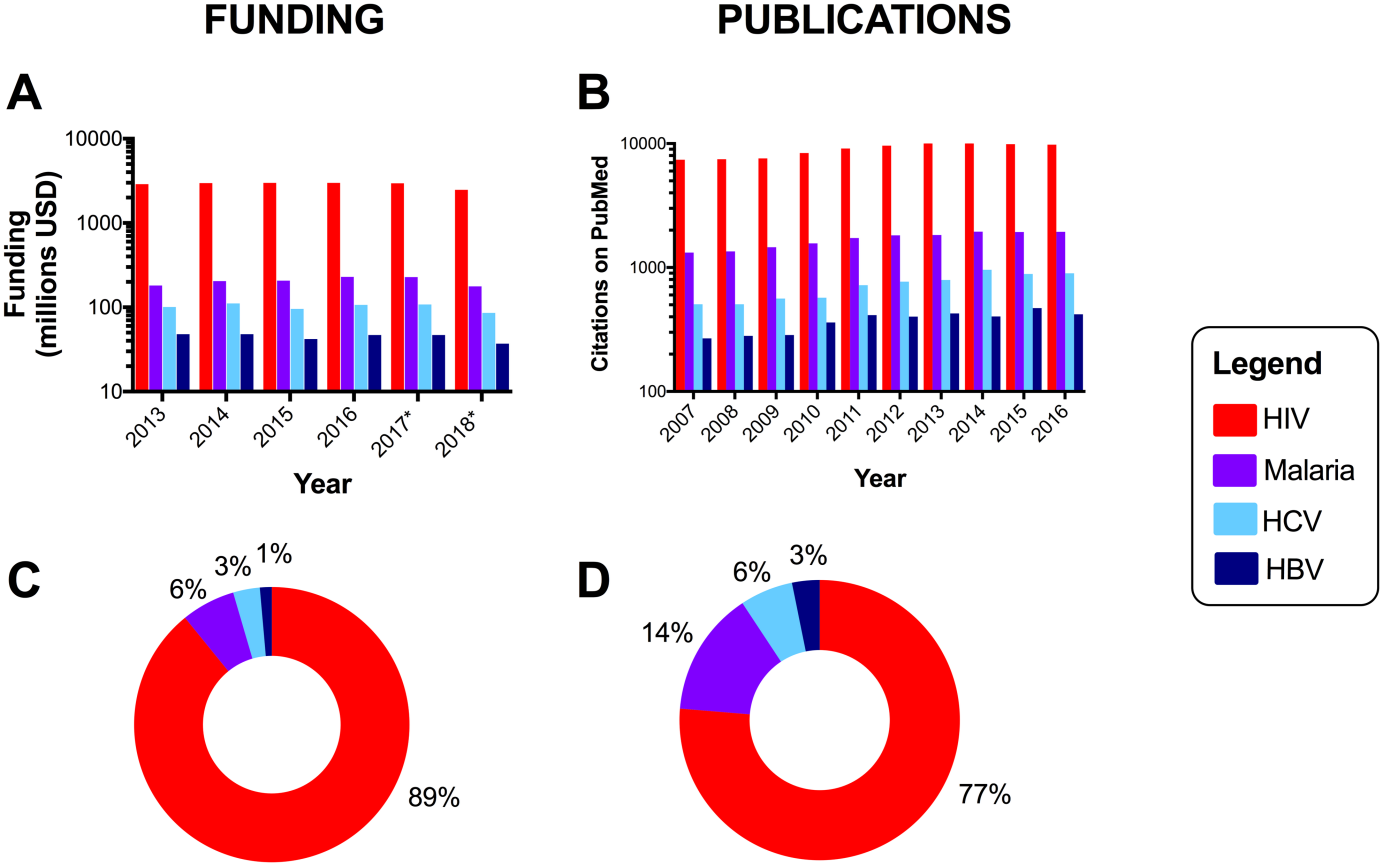
Resource gap in research funding allocations and academic publications for hepatitis B virus (HBV), hepatitis C virus (HCV), HIV, and malaria. Panels A/C: funding data from the United States National Institutes for Health (NIH) estimated funding for research, condition, and disease categories 2013–2018 (*projected figures for 2017 and 2018), available at https://report.nih.gov/categorical_spending.aspx, downloaded June 2017. For the projected funding allocation for 2018, HCV will receive 2.3-fold HBV funding, malaria 4.8-fold, and HIV 66.8-fold. Research into “malaria” and “malaria vaccine” are subdivided in the source data set but have been pooled in this graphic. Panels B/D: We recorded the number of publications listed on NCBI PubMed based on the search terms “HIV,” “HBV,” “HCV,” and “malaria” for each year from 2007–2016. Example search string for HBV publications in 2016: (HBV[Title]) AND ("2016/01/01"[Date—Publication]: "2016/12/31"[Date—Publication]). Data are represented as absolute numbers (panels A and B) and the proportion of the whole (panels C and D). For hepatitis delta virus (HDV), funding allocation data are not available, and we identified <25 publications/year (range 7–23).

Factors contributing to HBV neglect
• **Stigma and discrimination** leading to lack of patient voice (S1 Supporting Information; cases 5, 6, 7) [9].
• **Silent infection**, which may never be diagnosed and is not apparent to onlookers (contributes to large pool of undiagnosed infection).
• **Poverty**, leading to lack of patient voice, lack of public profile, and underrepresentation (S1 Supporting Information; cases 4, 5, 7).
• **Complacency** that ongoing deployment of existing resources and approaches (e.g., suppressive antiviral therapy and vaccination) is sufficient to bring about elimination [10].
• **High burden in low-/middle-income countries** [6], where investment is not a priority.
• **Lack of public/media representation**; no “high profile” cases.
• **HBV is “eclipsed” by higher profile infections** such as HIV and malaria.
• **Poor education and knowledge** among patients, the public, and healthcare workers (S1 Supporting Information; cases 6, 8, 9) including underrecognition of the global burden of infection.
• **Lack of existing investment** [11,12] contributing to a cycle of underinvestment (Fig 2).
• **Lack of development of infrastructure** through which to provide education, prevention, diagnosis, and treatment and as a way to collect robust data.
• **Poor-quality data** (poor understanding of epidemiology and risk factors, little recognition of the impact of stigma, lack of assessment regarding feasibility of interventions, etc.). (S1 Supporting Information; cases 7, 8)
• **Lack of major dedicated funding agencies**.

### NTDs “primarily affect populations living in tropical and subtropical areas”

Although HBV is endemic globally, the bulk of morbidity and mortality is now borne by low-/middle-income countries in tropical and subtropical regions [6,13]. In Africa, many populations are particularly vulnerable due to coendemic HIV infection and other coinfections, host and viral genetic factors, poverty, and lack of education and infrastructure [7]. In this setting, HBV has been eclipsed by the more acute and tangible health crisis of human immunodeficiency virus (HIV); only now in the era of antiretroviral therapy (ART) is it reemerging as a visible threat [S1 Supporting Information; case 2]. One illustration of this shift is the increase in deaths from HBV-related liver cancer over time that contrasts a reduction in AIDS deaths [14].

### NTDs “disproportionately affect populations living in poverty and cause…morbidity and mortality, including stigma and discrimination”

HBV is part of a cycle of poverty, with a high burden of morbidity and mortality in young adults. The economic burden on individual families can be particularly catastrophic in low- and middle-income settings [15], although robust data are lacking for Africa. In resource-poor settings, lack of education and scarce healthcare resources impinge on successful diagnosis and monitoring as well as failure to control symptoms where relevant. Stigma and discrimination are often invisible but can be potent and highly relevant challenges to the success of scaling up interventions for prevention, diagnosis, and treatment [9] [S1 Supporting Information; cases 1, 4, 5, 6, 7, 9].

### NTDs are “immediately amenable to broad control, elimination, or eradication by applying…public health strategies”

**Fig 3:**
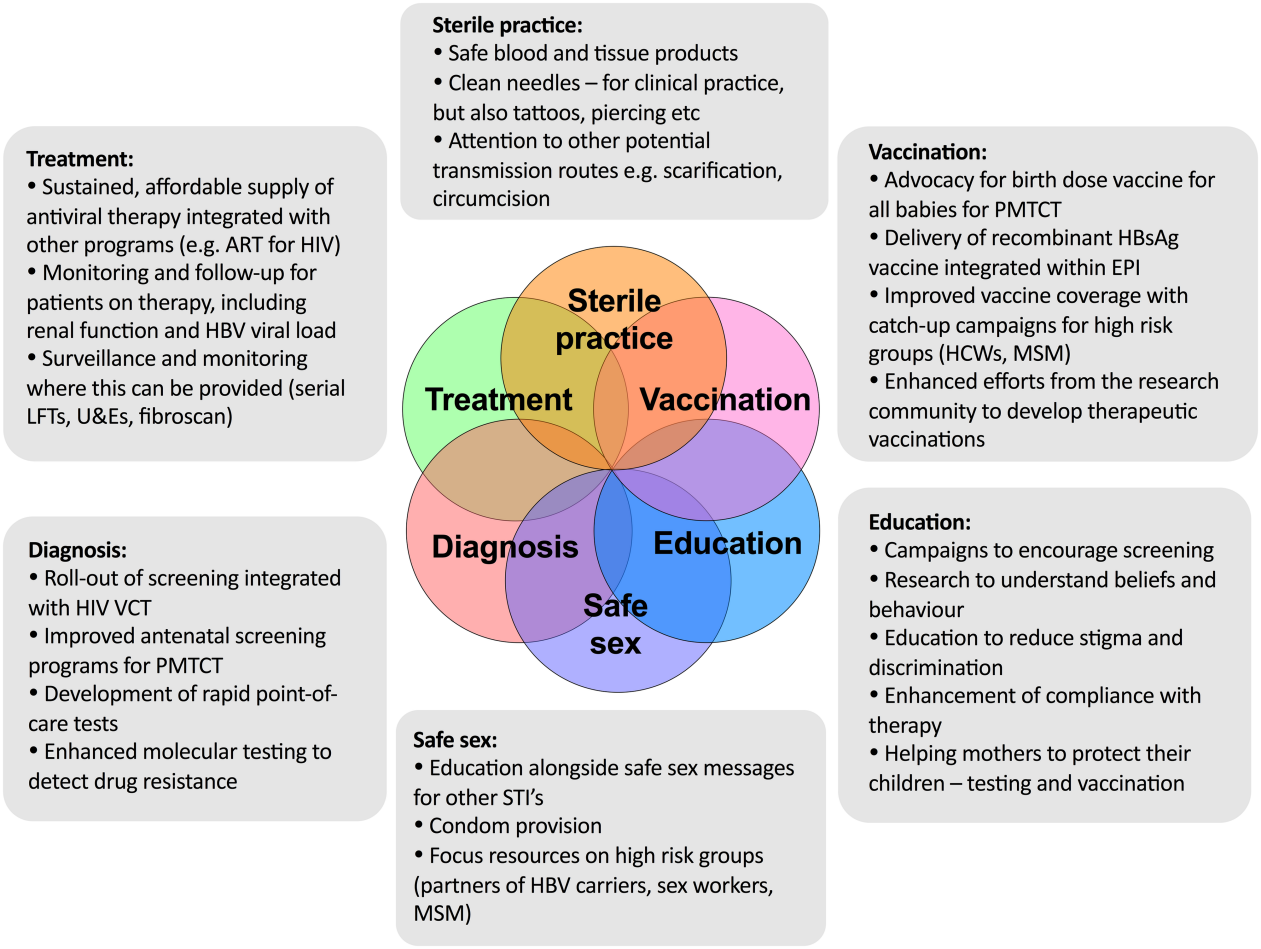
A package of interventions to move towards elimination of hepatitis B virus (HBV) infection as a public health threat. Suggested measures are aligned with WHO interventions for neglected tropical diseases (NTDs).

We already have an armamentarium of strategies with which to tackle HBV prevention and treatment (Fig 3). In order to be widely and robustly deployed, these approaches should interlink with existing resources and infrastructure wherever possible [S1 Supporting Information; case 2].

### NTDs are “relatively neglected by research—i.e., resource allocation is not commensurate with the magnitude of the problem”

Compared with other blood-borne viruses, namely, HIV and hepatitis C virus, which infect substantially lower numbers [7], HBV has attracted far fewer research resources, and this gap may actually be widening over time [11,12] (Fig 2). A recent report of infectious diseases research investment in United Kingdom institutions documents that 0.7% of total expenditure is for HBV, compared to 3.0% for HCV, 13.9% for malaria, and 17.5% for HIV [12]. HBV mortality (887,000 deaths/year [3]) is now twice that of malaria (429,000 deaths/year [16]), but malaria receives nearly 5-fold more funding (Fig 2). Hepatitis delta virus (HDV), a copassenger with HBV, can drive aggressive liver disease but is almost completely overlooked in terms of resource allocation. Moreover, development of clinical programs for hepatitis testing and treatment are fragmented in comparison to the progressive infrastructure that has emerged to tackle HIV [S1 Supporting Information; case 7].

## Recommendations based on NTD framework

Even for an organism that is not officially recognized as an NTD, there is much to be learnt from the NTD paradigm that could accelerate progress in tackling HBV. The ethos of combining several public health strategies and integrating care for different diseases is captured by the approach advocated for NTDs [4] and is also a helpful strategy for HBV. Particularly in the African subcontinent, where other NTD models have had significant impact [17], using this framework for HBV could promote awareness, leverage advocacy and resources, and promote integration of HBV prevention and treatment into existing HIV infrastructure [5].

In the following section, we use suggested interventions for NTDs to discuss briefly how these are pertinent to reducing—and ultimately eliminating—HBV infection as a public health threat.

### “Intensified case management”

Based on the significant numbers of individuals lost at every step of the “cascade” from diagnosis through to successful treatment and prevention (Fig 1), enhanced efforts are needed to promote linkage through care pathways. Enhanced HBV testing is crucial to facilitate entry into clinical care, allowing treatment to reduce the risk of onward spread, including underpinning PMTCT [S1 Supporting Information; case 8]. Initially, this may rely on using existing diagnostic platforms (based on serology), but investment is required in developing and rolling out new approaches, including molecular testing strategies that are more sensitive, provide enhanced data (e.g., detection of drug resistance), and are fast enough to enable point-of-care testing. This can often be transferred from technology that has been initially developed for the diagnosis of other diseases. Definitive curative therapy for HBV remains a crucial aspiration, as elimination using existing strategies is not realistic during the time frames set by SDGs [10]. Specifically, a therapeutic vaccine harnessing successful immune responses to boost immune clearance would provide a huge leap forward in tackling the existing burden of chronic infection [18].

The role and significance of stigma associated with HBV infection in Africa is largely unreported in the literature. However, individual testimony leaves no doubt that this is a significant barrier to diagnosis and clinical care [S1 Supporting Information; cases 5, 6]. Gaining a better understanding of the extent and nature of stigma and discrimination in different populations is a crucial first step, in parallel with enhanced efforts to educate patients, health care workers, and the public.

### “Preventive chemotherapy”

Although antiviral therapy for HBV is generally regarded as treatment rather than prevention, in the majority of cases, it renders individuals aviremic, preventing onward transmission. Antiviral therapy for HBV (Table 1) should be made accessible, ideally capitalizing on the supply chains and distribution infrastructure that have been developed for HIV (and/or other prevalent infections, such as tuberculosis and malaria) [5]. Research efforts are still required to identify prognostic factors that predict differential response to therapy and allow tailoring of care.

PMTCT can progressively become a realistic goal by expanding access to antenatal diagnostics, simple treatment interventions such as maternal tenofovir during trimester 3, and HBV vaccination for all babies, with the first dose delivered at birth [8]. Vaccination remains a cornerstone of prevention, but more work is needed to investigate the most effective catch-up immunization strategies to reduce the burden of HBV infection at a population level [S1 Supporting Information; cases 3, 4, 8].

### “Sanitation and hygiene”

Although this category of interventions is conventionally applied to reducing food- and waterborne infections, we here broaden our interpretation to include other aspects of prophylaxis. Safety and security of medical supplies has increasingly improved to reduce nosocomial transmission of blood-borne viruses over recent decades [S1 Supporting Information; case 3]. However, sterile practices need to be more widely promoted and guaranteed to assure the safety of other procedures such as scarification, tattoos, piercings, and circumcision that may occur in community settings. Provision of condoms alongside education regarding safe sex, particularly for high-risk groups such as sex workers and men who have sex with men, is another important strategy for prevention.

## Conclusions

Elimination of HBV infection has gained status within international health and development agendas but is a complex clinical and public health challenge that currently lacks proportionate multilateral commitment from pharma, government, commissioners, funders, and the research community. The many parallels with other NTDs are clearly exemplified by vulnerable populations of the African subcontinent. By viewing HBV—as well as its partner in coinfection, HDV—within the NTD framework, we can improve approaches to reducing the burden of disease and move towards eventual elimination.

## Supporting information

S1 Supporting Information This document contains supporting information to corroborate the view that hepatitis B virus (HBV) can helpfully be represented within the framework set out for neglected tropical diseases (NTDs) by the World Health Organization (WHO) [1]. This is in line with aims stated within Sustainable Development Goals (SDGs) [2]. Complementary evidence gathered from patients, researchers, and healthcare workers from different locations in Africa illustrates the ways in which HBV infection meets the criteria for NTDs. These scenarios (designated cases 1 to 9 and presented geographically in order from south to north) contribute important insights into how the NTD paradigm can be helpful in informing strategies to improve diagnosis, treatment, and prevention of HBV infection, with the ultimate goal of eliminating infection as a public health threat.
(PDF)
