# Supplementary material for: Hepatitis B virus infection as a neglected tropical disease
Source: PLoS Negl Trop Dis. 2017 Oct 5;11(10):e0005842. doi: 10.1371/journal.pntd.0005842 (PMC5628785; doi:10.1371/journal.pntd.0005842)
Supplement: S1 Supporting Information — This is in line with aims stated within Sustainable Development Goals (SDGs) [2]. Complementary evidence gathered from patients, researchers, and healthcare workers from different locations in Africa illustrates the ways in which HBV infection meets the criteria for NTDs. These scenarios (designated cases 1 to 9 and presented geographically in order from south to north) contribute important insights into how the NTD paradigm can be helpful in informing strategies to improve diagnosis, treatment, and prevention of HBV infection, with the ultimate goal of eliminating infection as a public health threat. (PDF) [file pntd.0005842.s001.pdf]

Supporting Information File

Hepatitis B Virus (HBV) Infection as a Neglected Tropical Disease (NTD)

Geraldine A O'Hara, Anna L McNaughton, Tongai Maponga, Pieter Jooste, Ponsiano Ocama, Roma Chilengi, Jolynne Mokaya, Mitchell I Liyayi, Tabitha Wachira, David M Gikungi, Lela Burbridge, Denise O'Donnell, Connie S Akiror, Derek Sloan, Judith Torimiro, Louis Marie Yindom, Robert Walton, Monique Andersson, Kevin Marsh, Rob Newton, Philippa C Matthews

Background

This document contains supporting information to corroborate our view that Hepatitis B Virus (HBV) can helpfully be represented within the framework set out for Neglected Tropical Diseases (NTDs) by the World Health Organization (WHO) [1]. This is in line with aims stated within United Nations Sustainable Development Goals [2].

We here provide complementary evidence from different locations in Africa to illustrate the ways in which HBV infection meets the criteria for NTDs. These scenarios, labelled Cases 1 to 9 (C1 - C9), are presented by geography from South to North. They contribute important insights into how the NTD paradigm can be helpful in informing strategies to improve diagnosis, treatment and prevention of HBV infection, with the ultimate goal of eliminating infection as a public health threat.

The resources provided here document real experiences of HBV from patients, healthcare workers and laboratory staff from urban and rural settings. Although some of them contain references to published data, the primary aim is to capture experience and opinion which are often not reflected in conventional academic publications. This reflects the true impact of HBV infection, and the challenges associated with reducing the burden of cases, ranging from insights into its close relationship with poverty, to the stigma and discrimination faced by individuals with the infection, through to the first-hand experience of clinicians and laboratory staff who seek - on a daily basis - to improve the standards of care they can offer to their patients and their communities. Alongside the strong political and strategic arguments that can be made for HBV advocacy, these real life voices represent a unique personal angle.

The case histories are all accurate first-hand representations of true events, but we have changed or omitted certain details in order to protect the anonymity of individuals concerned.

WHO criteria for NTDs

- NTDs 'primarily affect populations living in tropical and sub-tropical areas'.
- NTDs 'disproportionately affect populations living in poverty; and cause important morbidity and mortality - including stigma and discrimination'.
- NTDs are 'immediately amenable to broad control, elimination or eradication by applying...public health strategies'.
- NTDs are 'relatively neglected by research – i.e. resource allocation is not commensurate with the magnitude of the problem'.

Other characteristics of NTDS

- NTDs are often 'hidden and silent' – individuals affected have little political voice.
- NTDs have economic consequences ranging from potentially crippling costs to individuals and families, to imposing barriers upon socioeconomic development.

Strategies for tackling NTDs that are relevant to HBV

- Implementation of a combination of several public health approaches that can be delivered locally, and often in combination with infrastructure used to tackle other diseases.
- Provision of preventive chemotherapy.
- Intensification of case management.
- Improvements in sanitation and hygiene (applied broadly to HBV in reflecting improvements in sterile practice).

C1 | **Case 1:** Cape Town **South Africa**

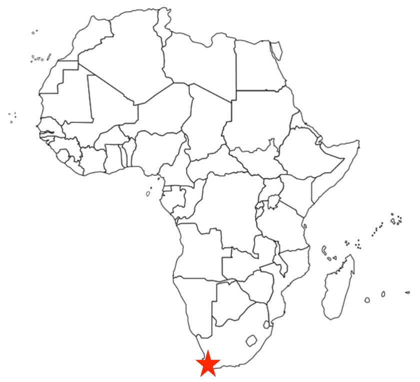

Relevance to WHO NTD framework

- HBV infection is associated with 'important morbidity and mortality' and is associated with high economic costs for individuals, families and society.
- The morbidity and burden of disease can be diminished through improvements in prevention, diagnosis and treatment.

Morbidity and mortality from HBV

A 24-year old man was admitted to hospital after reporting haematemesis. He was a college student and a teetotaller with no family history of liver disease.

CT imaging of the abdomen revealed a shrunken liver, ascites and multiple liver nodules. In combination with a raised serum alpha-fetoprotein (AFP) of >15000 µg/L, this suggested a diagnosis of hepatocellular carcinoma (HCC) with underlying cirrhosis. A serology profile was positive for HBV, but he was negative for HCV and HIV. He died 20 days after presentation to hospital.

Due to this patient's presentation with HCC and infection with HBV, his family members were subsequently tested. Chronic HBV infection was detected in two siblings, who have since been placed on antiviral therapy and on-going HCC surveillance. His mother was HBV-exposed but had cleared the virus.

This is a typical example of many young and economically-productive/promising patients who present with HCC in South Africa. These patients frequently present with advanced HCC arising from undiagnosed and untreated chronic HBV infection. Timely diagnosis of HBV infection remains a rarity. Many HBV-infected patients remain undiagnosed and at risk of developing HCC.

C2 **Case 2:**
Kimberley
South Africa

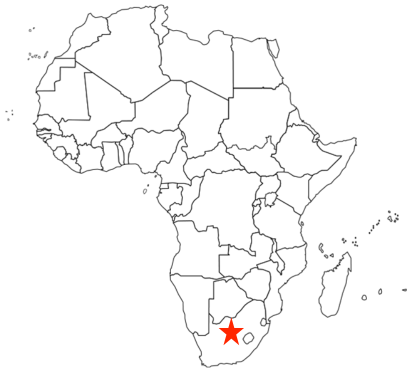

Relevance to WHO NTD framework

- HBV infection is silent – both clinically, where it can be asymptomatic until the very late consequences of chronic infection, and as a result of its niche in vulnerable populations with little voice.
- There is potential for HBV to be effectively tackled through existing infrastructure; in this case diagnosis and management is nested within HIV services.

A case of paediatric HIV/HBV coinfection

A ten year old girl presented to the Paediatric Clinic, referred with a positive HIV test (CD4+ T cell count 375 cells / mm^3 (20.8%) and HIV RNA viral load $>10^5$ copies/ml). She was assessed as WHO HIV infection Stage 1 with generalized lymphadenopathy and a BMI of 15 kg/m^2.

She was born in another region of South Africa, and no history was available regarding any perinatal prevention of mother to child transmission interventions. However, her records indicate that she had received three doses of HBV vaccine as per WHO EPI recommendations. Her mother was unwell, and the child had recently been taken into the care of another relative.

She was started on ART (Abacavir, Lamuvidine and Efavirenz) without any side effects. Her CD4+ T cell count and HIV viral load were repeated every 6 months but in spite of repeated counselling on adherence her HIV viral load persisted at $>10^4$ copies/ml. A year later, as a result of introducing HBV screening for all HIV-positive children [3], she was found to be HBsAg positive. HBeAg was also positive and her HBV DNA viral load was 36 x 10^6 IU/ml. Her liver function was normal and she has no symptoms to suggest chronic liver disease. The child's mother was HBsAg negative.

On the basis of a high HBV viral load despite lamivudine, and failure of HIV suppression, her ART was changed to tenofovir, emtricitabine and ritonavir boosted lopinavir. Following this, her HIV viral load became suppressed below the limits of detection for the first time, CD4+ T cell count improved to 863 cells/mm^3 and BMI improved to 17.4 kg/m^2.

This case illustrates the importance of routine screening for HBV, even without a family history of infection, and in the absence clinical evidence of liver disease. HBV viral load testing and a choice of appropriate therapeutic agents can be provided from within the infrastructure currently available for HIV.

C3

Case 3:
Lusaka
Zambia

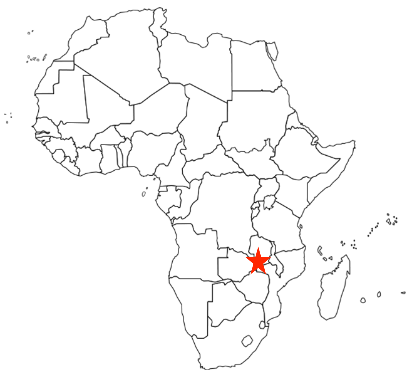

Relevance to WHO NTD framework

- HBV infection disproportionately affects vulnerable people living in poverty.
- NTDs can be tackled through implementation of a combination of several public health approaches that can be delivered locally, and integrated within other services.

Clinical experience from Zambia

(i) Relationship between HBV, poverty and morbidity

There is a huge overlap between high HIV prevalence, poor economic status, weak health systems, and HBV; the lack of resources makes it difficult to optimise prevention and control measures. HIV–HBV coinfected individuals may also be at risk in other ways; for example, they are more likely to have hazardous alcohol consumption [4]. According to preliminary data from the Zambia Population-Based HIV Impact Assessment (ZamPHIA) study [5], prevalence of HBV infection is higher among HIV-positive than HIV-negative adults (7.1% vs. 5.4%, respectively) and children (5.2% vs. 1.3% respectively).

(ii) Failure of current HBV vaccination schedule

While HBV is integrated as part of the pentavalent vaccine in Zambia delivered at 6, 10 and 14 weeks, the coverage declines with each consecutive dose. According to the 2013 Demographic Health Survey, coverage of the first dose was 79%, which reduced to 72% for the third dose [6]. No programme is in place to cover special populations at increased risk, such as health care workers and incarcerated individuals within correctional facilities.

While the prevention of mother to child transmission (PMTCT) of HIV is being scaled up, no systematic screening for HBV is in place and birth dose of the HBV vaccine is rarely provided. In contrast, screening for syphilis and HIV is hugely successful; this infrastructure could be a great opportunity to develop HBV screening and care, including vaccine doses for new-born babies.

(iii) Failure of assessment and treatment for individuals with chronic HBV infection

In Zambia, the National Blood donation system screens all blood donors. “We have raw data showing that we discard more much more blood because of HBV than HIV; and yet no linkage to care is provided for those found to be HBsAg-positive.”

C4 **Case 4:** Blantyre **Malawi**

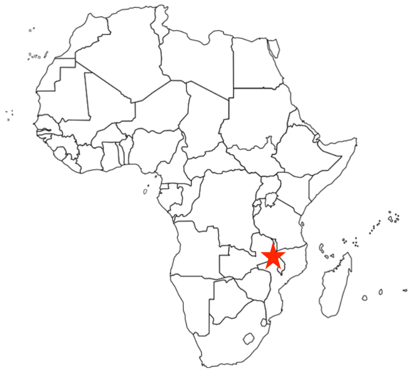

Relevance to WHO NTD framework

- Resources are constrained in comparison to those deployed for other infections; access to diagnosis, monitoring, treatment and prevention for HBV infection lags far behind that provided by HIV programmes.

Clinical experience from Malawi

Accurate data on national incidence and prevalence of hepatitis B in Malawi are lacking, however clinicians are familiar with the clinical consequences of chronic infection, as illustrated by the following case.

A 22-year-old man presented to hospital in Blantyre with sudden onset vomiting of blood. He had reduced consciousness, tachycardia and hypotension at presentation, though was successfully resuscitated with intravenous fluid, and blood donated by a family member.

An endoscopy revealed oesophageal varices which were banded, and a liver ultrasound indicated cirrhosis. The patient was commenced on propranolol to reduce the risk of subsequent gastrointestinal bleeding. Full hepatitis serology was unavailable, but HBsAg screening was available via the transfusion laboratory and was positive. An HIV test was negative.

Whilst anti-viral drugs with activity against hepatitis B were available (lamivudine and tenofovir) in fixed dose combination tablets via the HIV clinic, these were not accessible for patients with HBV monoinfection. The patient resorted to purchase of lamivudine on private prescription, noting that “I would have got treatment for free if I had HIV, but cannot find the drugs I need for hepatitis B, even though it is the same medicine”.

Although vaccination of household contacts was recommended, staff for contact tracing and supplies of the hepatitis B vaccine in public hospitals were lacking.

C5 | **Case 5:** Kilifi **Kenya**

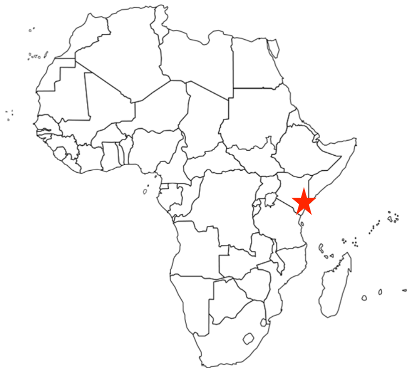

Relevance to WHO NTD framework

- These testimonies highlight the extent to which the morbidity of HBV infection arises as a consequence of poverty; this is significant for patients, but also has substantial impact for their extended families.
- Poverty can also be a barrier to prevention; for example, healthcare workers are unable to afford vaccination.

Healthcare workers and patients discuss the socio-economic impact of an HBV diagnosis

"Most of the patients with HBV infection only come to the hospital when they are extremely sick. The reason they come late is because they do not have money to seek medical help early enough and stay at home, hoping that they will get better. By the time they present to the hospital, they are in very bad shape and most of them die in hospital. Some of them die at home."

''A lot of patients with HBV infection present with symptoms suggestive of malaria or other commonly experienced diseases in the community. Only after they are treated for these conditions, and are not getting better, is an HBsAg test is done. The lack of proper investigations is mainly because the patient or their relatives lack the money to do the required investigations, or the facility is not capable of doing the necessary tests.''

"My cousin started complaining of stomach pain and thereafter started vomiting blood. He was diagnosed with HBV and died a few weeks later. My aunt was then tested and found positive. She goes for her medications weekly, but the cost of managing the condition is expensive and it is evident it has affected her lifestyle."

"My husband was taking his HBV medicines, though sometimes he missed a week or so when we could not afford to buy them. More recently, we have not been able to buy medicines and his condition is getting worse. Some health workers seem not to put that into consideration. Some scolded my husband for not taking his medicines and this made me feel so helpless as we have tried to sell our home produce and animals, but the burden continues to be heavier. I spend most of my time in the hospital with my husband and I have left my children in the care of their grandparents. I am worried that they may not have enough to eat as I am the breadwinner."

"Not all health care professionals working in public hospitals have been vaccinated against hepatitis B – the vaccine is not available in some public hospitals, so you have to go to a private hospital to get vaccinated and pay for it from your own pocket, which is very expensive.''

C6

Case 6:
Multiple locations
Kenya & Uganda

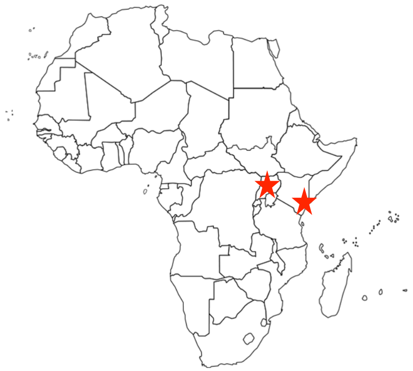

Relevance to WHO NTD framework

- HBV infection can be highly stigmatised. Lack of education and information are barriers to care.
- The burden from stigma and discrimination may fall disproportionately on girls and women within the community.

Young adults report stigma associated with HBV infection

“For healthcare professionals, those infected with HBV are isolated by their colleagues. No-one wants to go where they have gone. This also escalates up to where they stay, because people do not want their children near them.”

“For patients with hepatitis infection, some of them believe that they have been bewitched and start taking traditional medicines. They come to the hospital when they are jaundiced and in very bad shape.’’

“In our culture what happens to one person becomes the concern of the whole family and the community. Having a Hep B diagnosis will bring shame to one’s family. This can cost family members jobs and school places.”

“Hepatitis B is associated with HIV/AIDS, which means having a diagnosis comes with stigma and discrimination. This starts a trend of finger pointing, and slowly results in isolation. Although there has been a lot of work done to educate people about HIV, there has not been enough work to educate people about Hepatitis B.”

“Fear of disclosing the diagnosis is a big barrier to the patient accessing treatment. There is likely to be a danger of coinfections that increases fear about getting tested.”

“Mothers with HBV infection would be very keen to seek vaccination for their babies, but those most affected lack education to understand the importance of vaccinating a child at birth. There is little or even no information in areas worst affected - for example, people still believe the myth that vaccines pose more harm than good.”

“Health care workers are ignorant and don’t know how to handle or manage a situation when an individual discloses they are Hepatitis B positive but HIV negative. This confusion creates a situation where the health care workers are discriminating and stigmatising HBV patients due to lack of information.”

C7 | **Case 7:**
Entebbe and Kampala
Uganda

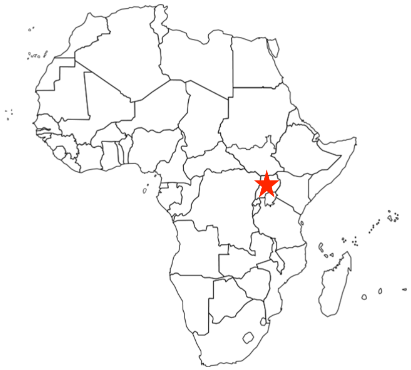

Relevance to WHO NTD framework

- Few facilities offer testing for HBV and for those diagnosed, linkage to care is lacking.
- There is a lack of guidance around diagnosis and monitoring.
- Antiviral medication is manufactured locally but is often inaccessible to those who need it due to cost or logistics.

Clinical experience from Uganda

The test currently available, and widely used, as a screening test for Hepatitis B infection is a rapid direct binding test for the detection of HBsAg (sandwich immunoassay). There is lack of testing for other Hepatitis B serum biomarkers (anti-HBc, anti-HBs etc). For patients that have received a diagnosis, monitoring and follow up is not done effectively because of shortages in supplies for laboratory testing of HBeAg and viral load.

In Uganda, there is one central government laboratory where HBV viral loads can now be tested. In practice, this facility is not yet routinely available and when it is, samples will have to be transported from all over the country to that laboratory. This has worked for HIV viral load testing, setting a previous precedent; we therefore have reason to be optimistic that it is feasible for HBV. One concern, however, is that the quality of the samples may have deteriorated by the time they reach the central laboratory. Currently, private testing facilities or NGOs are frequently used for viral load testing with costs of USD $50-60 per sample. This is unaffordable to our patients, so the majority of those who test HBsAg-positive do not get further assessment, and treatment is not taken up.

In Uganda, there is no algorithm for diagnosis of HBV, in contrast to the clear guidelines and policies for diagnosis and management of other conditions such as malaria, HIV and TB. As such, HBV diagnosis and monitoring has remained a grey area, and information passed onto patients is often unclear. This has created fear and increased the stigma for individuals with HBV infection. There is an urgent need to develop clear policies and guidelines that can be utilised by laboratory staff and health care workers on diagnosis and treatment.

Tenofovir monotherapy is available in Kampala. This is produced by CIPLA locally under Gilead voluntary licensing, and costs $7 per month of treatment. Despite the fact that this medicine is being manufactured locally, it is not yet reliably accessible for patients in rural areas. For these populations, this therapy can only be accessed through HIV care programmes, but these are lacking structure, manpower, training and guidelines with which to treat chronic HBV.

C8 | **Case 8:**
Yaounde
Cameroon

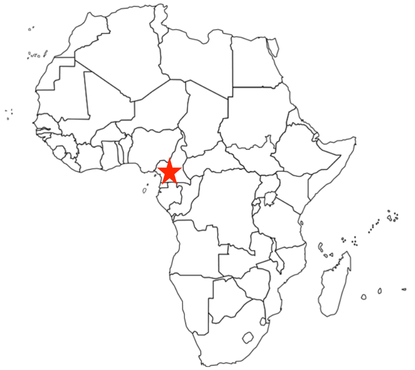

Relevance to WHO NTD framework

- Education and interventions for HBV PMTCT can be integrated into antenatal services already present for HIV.

Preventing Mother to Child Transmission (PMTCT)

Although there are some epidemiological data suggesting that HBV prevalence is 4-8% in the antenatal population, vertical transmission rates of HBV in Cameroon are unknown. Local data suggest that approximately 25% of pregnant women with HBV are HBeAg-positive [7], putting them at high risk of vertical transmission.

The vast majority of HBV-infected antenatal women are unaware of their HBV infection. This contrasts with HIV, for which most pregnant women now know their status. Although most pregnant women know that HBV vaccination can be used to prevent infection, they are mostly un-immunised themselves. Knowledge of other methods of prevention is poor, and many women are not aware that they can pass HBV infection on to their baby.

Education is needed to inform women of the potential for vertical transmission, to encourage them to seek diagnosis in pregnancy, get vaccinated themselves, and to access HBV vaccination for their babies. This can be integrated alongside existing education and interventions within HIV PMTCT programmes.

C9 | **Case 9:**
Multiple locations
The Gambia

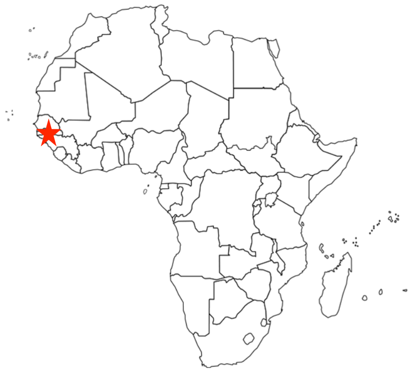

Relevance to WHO NTD framework

- Treatment options and palliative care for individuals with advanced liver disease are scarce, despite the availability of medication that can demonstrably improve quality of life.

Clinical experience from The Gambia

A fit-looking young man with a history of upper abdominal pain going back several months presented at a medical out-patient clinic. Amongst other investigations, an endoscopy was performed, revealing a dramatic picture of advanced oesophageal varices. He was subsequently found to be HBV infected.

Options for the treatment of advanced liver disease associated with HBV infection are limited in The Gambia, although the condition is not uncommon. This patient was started on medical treatment with propranolol, but died a few months later.

In another case, an otherwise-healthy patient in their 40s with hepatocellular carcinoma related to HBV infection presented at clinic looking pale and drawn. This is a very painful condition, due to stretching of the liver capsule. The only treatment to offer was regular, mild analgesia with paracetamol and codeine. However, on reassessment several weeks later, with symptoms now under control, he was a changed person, bounding into the out-patient clinic beaming with smiles.

Occasionally a physician has the opportunity to revolutionise a person's life. These occasions are relatively few, particularly when working in resource-poor settings with a limited therapeutic armamentarium. The care of people with terminal illness has been revolutionised by the hospice movement over the course of the last few decades by advances in symptom management. However, new ideas are sometimes slow to be adopted. In The Gambia, doctors are often reluctant to offer even simple analgesia such as paracetamol because of the perceived risk of liver damage. Opiates are frequently avoided altogether, or prescibed 'as required' rather than regularly, because of concerns about addiction or respiratory depression. Sociological factors may restrict progress in health care as significantly as economic constraints.

Hepatitis B Virus Infection as a Neglected Tropical Disease

References for supporting information file

[1] WHO. Neglected tropical diseases. 2017 http://www.who.int/neglected_diseases/diseases/en/

[2] Griggs D, et al. Policy: Sustainable development goals for people and planet. Nature 2013;495:305-307.

[3] Jooste P, et al. Screening, characterisation and prevention of Hepatitis B virus (HBV) co-infection in HIV-positive children in South Africa. J Clin Virol. 2016;85:71-74.

[4] Vinikoor M, et al. Impact of Antiretroviral Therapy on Liver Fibrosis Among Human Immunodeficiency Virus-Infected Adults With and Without HBV Coinfection in Zambia. Clin Infect Dis. 2017;64:1343-1349.

[5] Zambia Population-Based HIV Impact Assessment ZAMPHIA 2015-2016. http://phia.icap.columbia.edu/wp-content/uploads/2016/09/ZAMBIA-Factsheet.FIN_.pdf

[6] Zambia Demographic and Health Survey 2013-14 https://www.dhsprogram.com/pubs/pdf/FR304/FR304.pdf

[7] Fomulu N, et al. Prevalence, correlates and pattern of Hepatitis B among antenatal clinic attenders in Yaounde-Cameroon: is perinatal transmission of HBV neglected in Cameroon?
